# Mesoscopic Fluid-Particle Flow and Vortex Structural Transmission in a Submerged Entry Nozzle of Continuous Caster

**DOI:** 10.3390/ma15072510

**Published:** 2022-03-29

**Authors:** Peng Zhao, Rongxun Piao, Zongshu Zou

**Affiliations:** 1 School of Materials Science and Engineering, Shandong Jianzhu University, Jinan 250101, China; zhaopeng3@hotmail.com; 2 Department of Mechanical Engineering, Anhui University of Science and Technology, Huainan 232001, China; 3 School of Metallurgy, Northeastern University, Shenyang 110819, China; zouzs@mail.neu.edu.cn

**Keywords:** fluid-particle flow, submerged entry nozzle, continuous casting, lattice Boltzmann method, large eddy simulation, vortex structures

## Abstract

Understanding the essence of the flow oscillations within a submerged-entry nozzle (SEN) is essential to control flow patterns in the continuous casting mold and consequently increase the superficial quality of steel products. A numerical study of the mesoscopic fluid-particle flow in a bifurcated pool-type SEN under steady operating conditions is conducted using the lattice Boltzmann method (LBM) coupled with the large eddy simulation (LES) model. The accuracy of the model has been verified by comparing vortex structures and simulated velocities with published experimental values. The LBM modeling is also verified by comparing the “stair-step” jet patterns observed in the experiment. The geometrical parameters and operational conditions of physical experiments are reproduced in the simulations. By comparing the time-averaged velocities of Reynolds-averaged Navier–Stokes equations (RANS) with LBM models, transient mesoscopic fluid-particles and related vortex structures can be better reproduced within the SEN. The visualization of internal flow within the SEN is illustrated through the mass-less Discrete Phase Model (DPM) model. The trajectories show that the LBM–LES–DPM coupled model is good at predicting the transient vortical flow within the SEN. A large vortex is found inside the exit port and continuously changes in shape and size therein. The monitoring points and lines within the SEN are selected to illustrate the velocity variations and effective viscosity, which can reflect the oscillating characteristics even under stable operating conditions without changes at the exit from the SEN. Furthermore, the formation, development, diffusion, and dissipation of the vortex structures from the exit port of the SEN are also investigated using the *Q* criteria. The comparison of the power spectrum with high-frequency components along the exit port indicates that the flow oscillations must originate from within the SEN and are intensified in the exit port. The mesoscopic LBM model can replicate the fluid-particle flow and vortex structure transmission as well as their turbulence effects inside the SEN in detail.

## 1. Introduction

A common element in flow networks (industrial piping systems) is a T-junction that splits the flow into two nearly symmetric streams. What these systems often have in common is the presence of low-density particles or air bubbles, which get trapped at the junction, accumulate, and ultimately change the flow distribution. The unanticipated trapping mechanism can create malfunctions, and unexpected dangers in industrial environments [1]. The flow behavior presented inside the SEN has similarities to the fluid flow behavior in other engineering fields. One of the most significant challenges in continuous casting is to obtain clean steel. The quality of steel produced by continuous casting depends mainly on the characteristics of the liquid steel flow pattern within the mold. This pattern depends on the flow dynamics of the nozzle that is immersed in liquid steel. The behavior of the transient flow within the SEN has a determinant influence on the flow pattern, which was believed to be associated with the internal and superficial steel quality, such as its effects on asymmetrical defects such as slip cracking, pinholes, and blisters [2,3,4,5,6,7]. Consequently, these results show the importance of an accurate description of the behavior of the flow. Therefore, a better understanding of the flow structures and development of vortices within the SEN can be beneficial to quality control and improvements of the process such as reducing energy consumption and the cost of high-quality slabs and foundry alloy (silicon aluminum alloys) [8].

Since the effect of SEN flow on mold flow is difficult to detect under real operating conditions, experimental models have been applied to investigate the flow of steel in practice. As a supplement to experimental methods of investigation, the flow within the SEN also has been subject to numerical simulations. To reproduce the flow pattern in an SEN, the behavior of the fluid inside the SEN has been studied using the models based on the Reynolds-averaged Navier–Stokes equations (RANS), such as the *k*-*ε* turbulence model [9,10,11,12]: RANS models can reproduce time-averaged average flow patterns. The symmetrical flow inside the SEN is obtained when the velocity inlet is aligned perfectly with the SEN bore, however, they do not allow replication of transient dynamic flow behaviors. The observations in steel-making and physical simulations show that the transient behavior inside the SEN ports evolves with time. If gas bubbles or inclusions are introduced into SEN flow, the simulations will become significantly more complicated. Common to these works is the investigation of fundamental aspects that modify the flow pattern inside the mold. For example, Calderon-Ramos et al. [13] studied the effect of both the shape of the exit ports and the angle of inclination of the ports on symmetric jets. Similarly, Zhang et al. [14] studied the influence of the port angle and immersion depth of the SEN exit ports on the liquid steel flow pattern, which can reduce mold-level fluctuations.

Compared with the RANS model that provides time-averaged variables of turbulent flows, the LES model can provide more detailed instantaneous turbulence to capture small vortices better. In the LES model, large-scale eddies are solved directly with the filtered Navier–Stokes equations, while small-scale eddies are modeled as sub-grid scale grids. SEN flow and mold flow have been further investigated through the LES model on regular grids. Similarly, the presence of unsteady flow inside the SEN has also been described in previous work, allowing comparison of their results with physical experiments. For example, Real et al. [15] studied the periodic flow behavior inside the SEN using the LES model. Flow separation occurs in the upper region of SEN, where the area suddenly increases in channels. There are differences in the influence that each of these geometric properties has on the flow pattern of liquid steel inside the nozzle. Liu et al. [16] further developed computational fluid dynamics (CFD) methods by coupling them with LES models to simulate asymmetrical phenomena at both sides of the mold. Gonzalez-Trejo et al. [17] characterized the fluid dynamics of two separate SENs without exit ports, one with a well and the other without a bottom well, through LES with dynamic *k*-equation filtering. The model reproduced the dynamic nature of the internal flow pattern seen in physical experiments, however, only the bifurcation of the flow of steel in nozzle interior was examined and the effects of the port shape were not considered in their research.

Recently, the mesoscopic LBM model has been used to demonstrate micro-fluidics and complicated turbulent flows: this might be of interest to engineers running continuous casting operations. Pirker et al. [18] predicted the horizontal secondary vortices and bubble aggregation through the embedded lattice Boltzmann sub-region positioned in the bottom SEN region. The author [19,20] predicted the complicated flow pattern and coherent vortices inside a continuous casting mold. These studies have produced significant results, reflecting the potential advantages that predict local complicated flow behaviors. Flow structures play an essential role in complex flow regimes inside the SEN; however, previous work has paid little attention to the exploration of interior asymmetrical flow structure and their turbulent interaction mechanisms using the LBM–LES model inside the SEN.

The present work explores the fluid-particle flow and vortex structure transmission in jets that emerge from the nozzle ports as well as their turbulent effects. The effects of the tundish sliding nozzle and argon bubbles on liquid steel flow or any change in the operational parameters are not included. The bifurcated pool-type SEN and rectangular geometry of the SEN ports should be considered in this simulation. The fluid-particle flow within the SEN can be reproduced using the LBM model. The accuracy of the model is verified by comparing vortex structures and simulated velocities with results reported in the literature. The transmission of the vortex structures along the exit port of the SEN is also explored by the *Q*-criterion. The monitoring points and lines are finally selected to study asymmetrical turbulent flow at different positions of the exit port inside the SEN.

## 2. Model Formulation

### 2.1. Lattice Boltzmann Method

Lattice Boltzmann Method is given by [21]:

(1) $$f_{i}\left(x+c_{i},t+\delta t\right)-f_{i}(x,t)\;=\;\frac{1}{\tau_{\mathrm{LB}}}\left(f_{i}\left(x,t\right)-f_{i}{}^{\mathrm{eq}}\left(x,t\right)\right)+{\vec{F}}_{i}$$

where *c_i_* represents the discrete lattice velocities; *δ* denotes the time step; $f_{i}(x,t)$ is the discretized distribution functions; $\tau_{\mathrm{LB}}$ represents the dimensionless relaxation time; $\vec{F}$ is the gravitational force adopted into the LBM model; $f_{i}{}^{\mathrm{eq}}\left(x,t\right)$ is Maxwell distribution equilibrium function given by:

(2) $$f_{i}{}^{\mathrm{eq}}\left(x,t\right)=\rho w_{i}\left[1+\frac{c_{i}\cdot u}{C_{s}^{2}}+\frac{{\left(c_{i}\cdot u\right)}^{2}}{2C_{s}^{4}}-\frac{u^{2}}{2C_{s}^{2}}\right]$$

where $C_{s}$ is the sound speed, $C_{s}=1/\sqrt{3}$; *w_i_* is weight according to the lattice discretization, and *i* range from 0 to 18, as follows:

(3) $$w_{i}=\left\{\begin{array}{ll} \frac{1}{3} & i=0\\ \frac{1}{18} & i=1–6\\ \frac{1}{36} & i=7–8 \end{array}\right\}$$

The model D3*Q*19 is applied for the LBM model, and the collision operator employs the Bhatnagar-Gross-Krook (BGK) model with a single relaxation time [22].

The macroscopic quantities are calculated by taking the moments of distribution functions:

(4) $$\rho =\sum_{i}f_{i}$$

(5) $$\rho u=\sum_{i}c_{i}f_{i}$$

where *ρ* is the macroscopic density; *u* is the macroscopic velocity.

### 2.2. Large Eddy Simulation

The subgrid-scale stresses *τ_ij_* is defined by Smagorinsky subgrid model [23]:

(6) $$\tau_{ij}-\frac{1}{3}\delta_{ij}\tau_{kk}=-2\upsilon {\bar{S}}_{ij}$$

where $\delta_{ij}$ is the Kronecker delta function, the isotropic part of the Reynolds stress term $\tau_{kk}$ is included in the pressure term; the subscripts *i* and *j* represent three Cartesian directions.

${\bar{S}}_{ij}$ is expressed as the rate of large-scale strain tensor given by

(7) $${\bar{S}}_{ij}=\frac{1}{2}\left(\frac{\partial {\bar{u}}_{i}}{\partial x_{j}}+\frac{\partial {\bar{u}}_{j}}{\partial x_{i}}\right)$$

The viscosity υ in the Smagorinsky model is defined as:

(8) $$\upsilon =\upsilon_{0}+\upsilon_{\mathrm{eddy}}$$

where $\upsilon_{0}$ is the kinematic viscosity; $\upsilon_{\mathrm{eddy}}$ is eddy viscosity given by:

(9) $$\upsilon_{\mathrm{eddy}}=C\Delta^{2}\left|\bar{S}\right|$$

where Δ is expressed as the minimum size; *C* is the Smagorinsky constant, which depends on the size of the grid, and the value ranges between 0.1 and 0.2.

Relaxation time $\tau_{\mathrm{LB}}$ can be expressed as:

(10) $$\tau_{\mathrm{LB}}=3\left(\nu_{0}+C\Delta^{2}\left|\bar{S}\right|\right)+\frac{1}{2}$$

where $\left|\bar{S}\right|$ is the intensity of the local filtered stress tensor given by:

(11) $$\left|\bar{S}\right|=\frac{\sqrt{\upsilon_{0}^{2}+18C\Delta^{2}\sqrt{{\bar{\prod }}_{i,j}{\bar{\prod }}_{i,j}}}-\upsilon_{0}}{6C\Delta^{2}}$$

where ${\bar{\prod }}_{i,j}=\sum_{\alpha }c_{\alpha i}c_{\alpha j}\left|{\bar{f}}_{i}-{\bar{f}}_{i}^{\mathrm{eq}}\right|$ is the local non-equilibrium stress tensor.

## 3. Model Setup and Validation

### 3.1. Calculation Details

**Figure 1:**
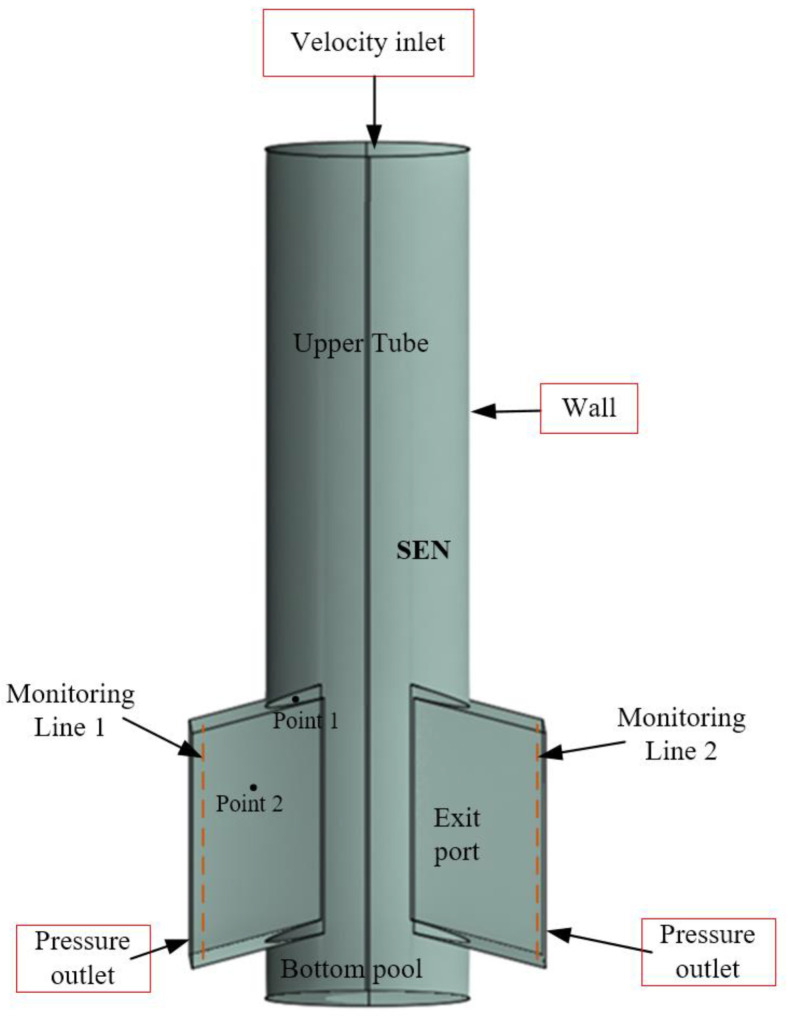
SEN geometry and two monitoring points.

The computational domain includes a bifurcated SEN nozzle with the bottom pool. The global computational domain comprises the incoming duct, the flow deflection region, the two opposing port openings, and part of the emerging confined jets. Table 1 lists the geometrical SEN, simulation conditions, and operating parameters considered in the simulation.The lattice spacing is set to 2 mm. This computational domain is discretized by approximately 500,000 elements in the LBM model. The time step for these simulations is 0.02 s. The resolved scale is 0.002 m. The starting time step for these simulations is 0.001 s. The inlet and outlet boundary conditions in the model are based on the bounce-back condition for the non-equilibrium portion of the distribution. The constant velocity inlet boundary condition is based on the casting speed at the inlet of SEN. The pressure outlet condition is applied in the exit outlet at both sides of the SEN. Figure 1 shows a schematic diagram of the geometry and boundary conditions as well as monitoring points and lines within the SEN considered in the study. 

### 3.2. Validation

The complex flow consists of multiple vortices in the mold. The behaviors of the jet emerging from the exit port of the SEN play an essential role in the flow pattern in the continuous casting mold. The jet vortices induced by the *Q*-criterion are investigated using the LBM model. The *Q*-criterion is given by [24]:

(12) $$Q=-\frac{1}{2}\frac{\partial u_{i}}{\partial x_{j}}\frac{\partial u_{j}}{\partial x_{i}}=\frac{1}{2}\left(\Omega_{ij}\Omega_{ij}-\Psi_{ij}\Psi_{ij}\right)>0$$

where $\Omega_{ij}$ and $\Psi_{ij}$ are the symmetric and anti-symmetric components of ∇u, respectively. Thus, the evolution of structures flow is deduced from the *Q*-criterion.

**Figure 2:**
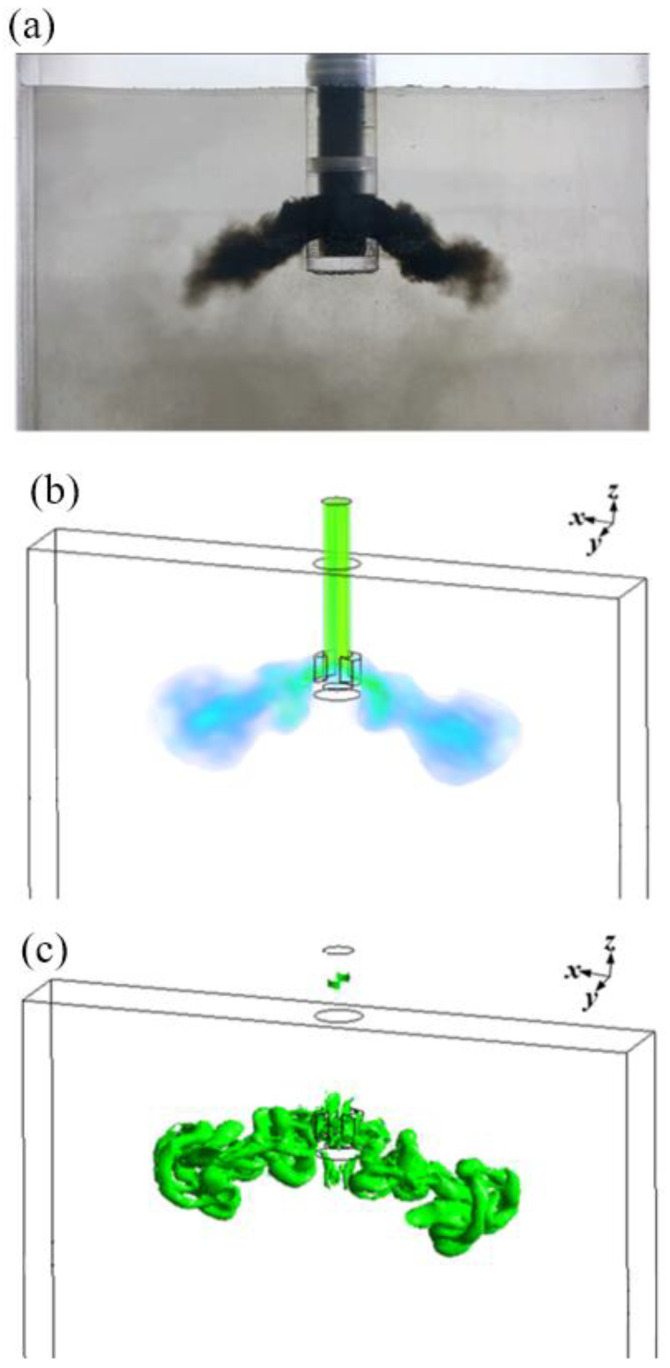
Comparison of the “stair-step” jet inside the mold under the same conditions through (**a**) the water experiment, (**b**) predicted velocity, and (**c**) vortex structures.

The models have been applied to a one-half scale water model for comparison, and the details of geometry and operating conditions are treated as being under the same conditions. Simulated velocity profiles are compared with previous experimental measurements. Figure 2a–c illustrates a comparison of the “stair-step” jet outside the SEN through (a) water experiment, (b) predicted velocity, and (c) vortex structures. The simulation results of the patterns and vortex structures of the jets also agree with the observations from water experiments, which reproduce the “stair-step” jets at two sides of the SEN when black ink is injected into the upper SEN tube. By comparing the simulation and the water experiment, the LBM model shows that it can be used to model the behavior of the oscillating jets within the SEN.

**Figure 3:**
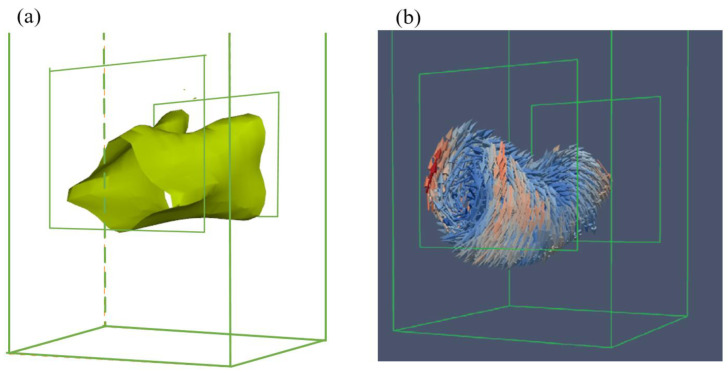
Comparison of (**a**) turbulent fluid flow pattern through the LBM model (left) with (**b**) vortex structure outlined by the fluid velocity vectors for Case A [17] (right) inside the NIP.

To verify the accuracy of the modeling the interior flow inside the SEN, the LBM model also has been applied to the water experiments for a comparison of experimental velocities and simulation results. Simulated velocity profiles are compared with those measured results from the water experiment under the same conditions, and the details of geometry and operating conditions are available in the literature [17]. Figure 3 illustrates a comparison of the turbulent fluid flow (left) through the LBM model with the vortex structures outlined by the fluid velocity vectors for Case A (right) inside the nozzle internal prototype (NIP). The NIP is used to evaluate the vortex distribution. As can be seen from the figures, there is a dominant vortex that occupies a large fraction of the volume of the nozzle. Figure 3a shows the vortex structure (isosurface of *v* = 1.2 m/s) in this simulation close to the exit port of the SEN. Vortex tube outlined by the velocity vectors is present in the same projection in Figure 3b. This surface outlines the shape of the vortex that can be produced inside the NIP. The comparison shows the vortex structures in this model are consistent with the vortex surface along with the velocity vectors in the previous simulation for Case A with the well pool. The LBM model can be validated to obtain internal flow and structure inside the SEN.

**Figure 4:**
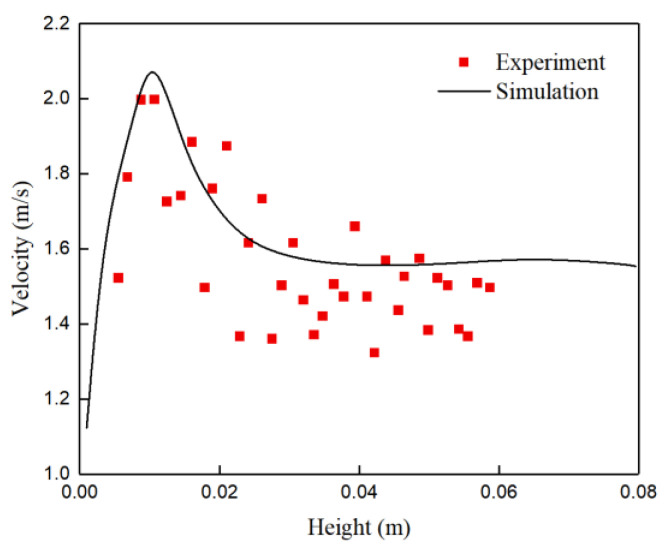
Comparison of experimental results for Case A [17] and predicted velocities prediction at the left-hand line of the SEN.

To compare the velocity of the flow inside the NIP, the velocity magnitudes along two straight lines from the physical model are available in the literature [17]. Figure 4 illustrates a comparison of experimental results along the path in Case A and simulated time-averaged velocities that measured three times under the same conditions. The measurements are undertaken along vertical lines close to the left side of the exit nozzle: these show the shape of the velocity profiles changes at different heights of the SEN. The velocity fluctuates significantly at the nozzle exit. The predicted velocity increases with the height of the exit port of the nozzle until it reaches a maximum value and then decreases with the height thereof. In the right-hand figure, the agreement between both methods is not as good as the velocity profile across the height of the exit port; however, the result reproduces the velocity profile as a function of SEN height, qualitative agreement is obtained.

## 4. Results and Discussion

### 4.1. Fluid-Particle Flow

**Figure 5:**
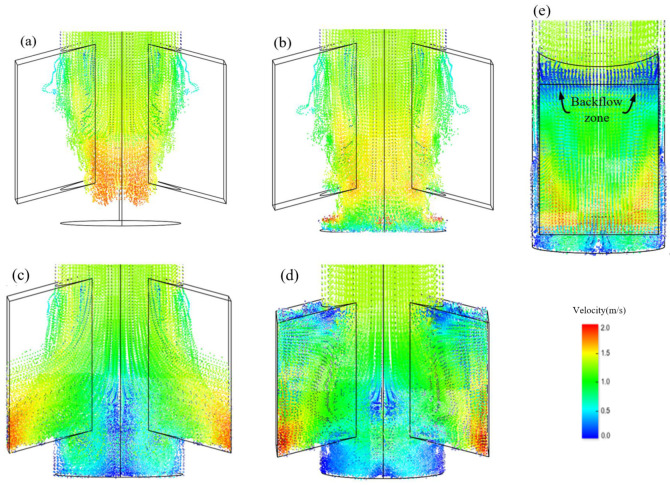
Transient evolution of mesoscopic fluid-particle flow inside the SEN at different times of (**a**) 0.1, (**b**) 0.2, (**c**) 0.25, (**d**) 0.3, and (**e**) 0.35 s.

The LBM model for the fluid-particle flow is also conducted in this simulation. To show the transient process of fluid-particle flow in the SEN under the steady-state conditions, Figure 5a–d show the fluid-particle flow patterns at 0.1, 0.2, 0.25, 0.3, and 0.35 s, respectively. The transient process of fluid-particles can be described as follows: transient fluid-particles emerge from the inlet of the SEN bore. The upper SEN pipe is filled with the fluid-particles; thereafter, the particle-based jet impinges onto the bottom of the SEN pool at a high velocity, and flow towards two sides of the SEN port. Meanwhile, the fluid-particles at the SEN pool interact with those from the upper tube; finally, the fluid-particles almost fill the entire the SEN port, meanwhile, backflow is also found in the upper region of the SEN port, as shown in Figure 5e. The fluid-particles at the SEN port interact with those within the backflow, resulting in more complex transient flow within the SEN. The results show that the mesoscopic LBM model can obtain the transient behavior of fluid-particle flow within the SEN in detail.

**Figure 6:**
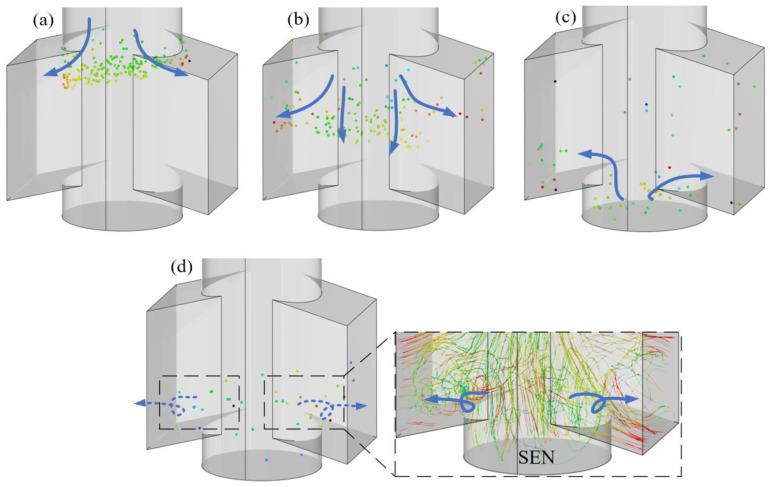
The evolution of passive particles flow and stream-tracers within the SEN at different stages: (**a**) corner separation, (**b**) central diffusion, (**c**) bottom diffusion, and (**d**) vortical flow.

Passive stream-tracers allow the simulation to track Lagrangian particles (mass-less) along the fluid, which is thus useful to better observe the streamlines lines or flow trajectories. The mass-less Discrete Phase Model (DPM) is used here to describe the vortical flow within the SEN. The visualization of internal flow within the SEN is illustrated in the following process (Figure 6a–d): Mass-less particles start to be separated at the corners between the upper tube and the exit port of the SEN. Then, some particles flow directly towards the exit port, while others move into the lower region until they impinge on the bottom wall of the SEN. These mass-less particles finally flow along the bottom region toward the exit port, accompanied by swirling particles-flowlines close to port of the SEN (see enlarged area Figure 6d). The trajectories show that the LBM–LES–DPM coupled model is good at predicting the transient vortical flow within the SEN because the inclusions are found dispersed at a fairly low volume fraction (usually less than 20%) in the steel through actual industrial experiment.

**Figure 7:**
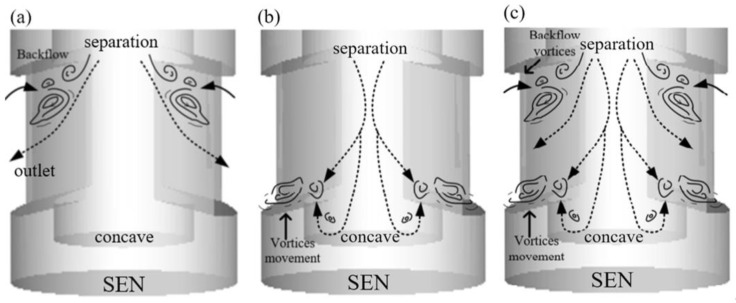
Schematic streamlines of the formation and development of internal flow corresponding coherent vortices inside the SEN at different stages: (**a**) corner separation, (**b**) bottom diffusion, and (**c**) vortical flow.

In order to better explain the mechanism of internal vortices structures inside SEN, Figure 7a–c schematically shows the formation of internal flow corresponding coherent vortices inside the SEN. The main processes are as follows: (a) Vortex structures are formed at upper region, meanwhile, they interact with the backflow zone at the upper region of the SEN; (b) vortex structures are concentrated near the concave shape, and they also interact with the incoming flow from the upper tube; (c) vortices structures from the upper region interact with those at the bottom concave, which can cause a more complex transient flow inside the SEN. In short, fluid separation occurs at the upper separation and the lower concave of the SEN. Vortex vortices split into small vortices and interact with each other, leading to the accumulation in these regions and intensifying the oscillating jets from the SEN port thereon.

**Figure 8:**
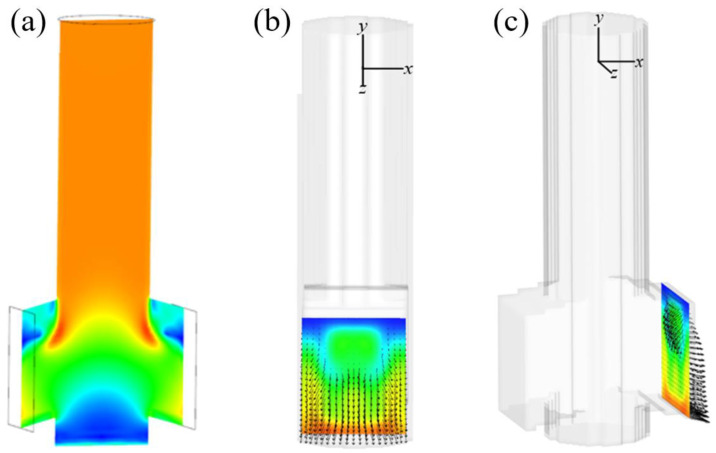
The time-averaged velocity contour and vectors at the exit port of the SEN from different views: (**a**) the middle plane *XZ*, (**b**) the plane *XY*, and (**c**) the exit port.

To quantify the time-averaged velocity of this mesoscopic fluid, Figure 8a illustrates the time-averaged velocity contour on the middle plane (*XZ*). The simulation results are measured three times with an interval of 10 s. These maps indicate that the flow patterns in both exit ports are identical. The symmetric flows on both sides of the SEN ports are present. Additionally, the flow velocity contour and vectors at the exit port of the SEN are also included in Figure 8b. The corresponding value of *X* for the plane is −0.03 m. Note that the area has a very low velocity along the upper edges of the exit ports, while fluid flow with high velocity is uniformly distributed at the bottom of the exit ports. A close inspection of the low-velocity vectors directed towards the interior at the upper SEN shows the existence of reverse flow (Figure 8c), which promotes the oscillation of the jet as it emerges from the exit port of the SEN.

**Figure 9:**
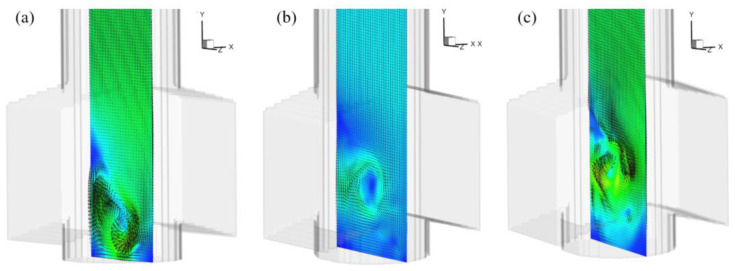
Transient vortex contours and trace streams on the center plane perpendicular to the exit port of the SEN at different times: (**a**) the large vortex appears at the bottom, and (**b**) the vortex gradually flows upward, (**c**) the vortex moves towards the middle output.

The better to show the evolution of flow structure at the exit port, Figure 9a–c shows transient vortex contour and trace streams perpendicular to the exit port of the SEN under the same condition. The simulation takes, as an example, the situation where a large vortex is generated inside the SEN with a well pool. The analysis of the simulation led us to the following observations: the big vortex located close to the bottom of the SEN is always present and is cylindrical, however, there is always a bias of the vortex axis with respect to the central plane of the SEN. The rotation axis of the large vortex is not substantially parallel to that of the exit ports. This large vortex oscillates continuously, changing in both shape and size.

**Figure 10:**
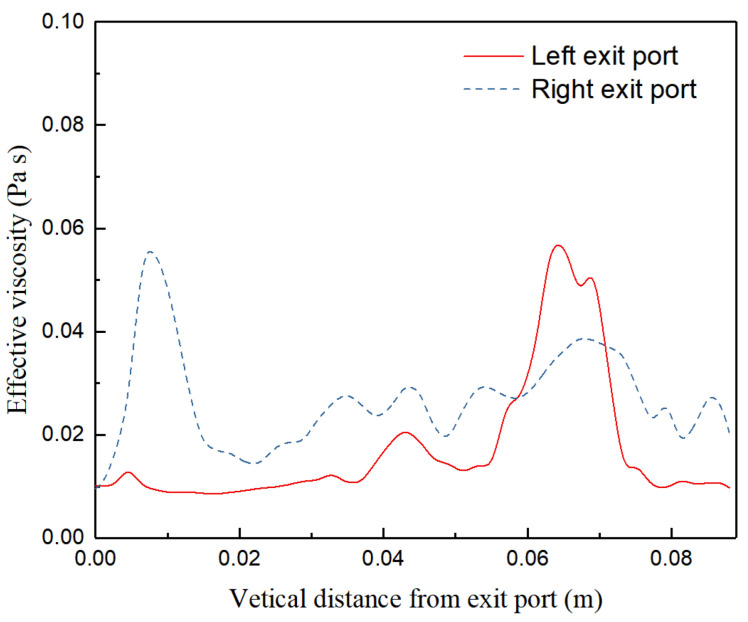
Asymmetric effective viscosities of the turbulent flow along the vertical monitoring lines close to the exit port of the SEN.

**Figure 11:**
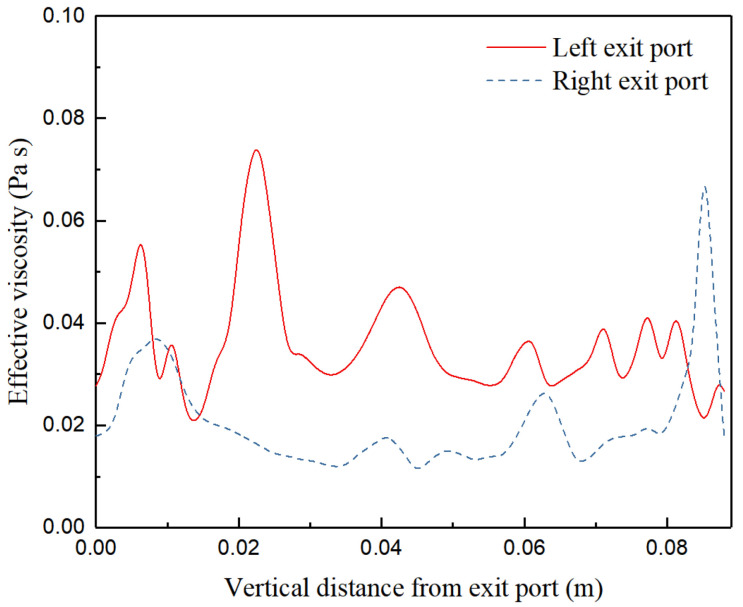
Mirror image of effective viscosities of the turbulent flow along the vertical monitoring lines close to the exit port of the SEN.

To quantify the turbulent flow close to the exit port, the effective viscosities (on the monitoring lines) at both sides of the exit points are investigated. The locations of the two reference lines are illustrated in Figure 1. Figure 10 and Figure 11 show the effective viscosities of the turbulent flow along the vertical monitoring lines close to exit port of the SEN at different times (Figure 1). From the figures, the turbulent viscosity of flow near the wall of the mold always exhibits fluctuation characteristics at both sides of the exit port. Figure 10 demonstrates the asymmetric viscosity at both sides, where the viscosity on the left upper port is higher than that on the right. Figure 11 shows a similar mirror image of the viscosity distribution, where the viscosity on the right upper port is higher than that on the left. It can be inferred that the viscosity change is related to the wobbling jet behavior, reflecting asymmetric changes in flow patterns from the exit port.

**Figure 12:**
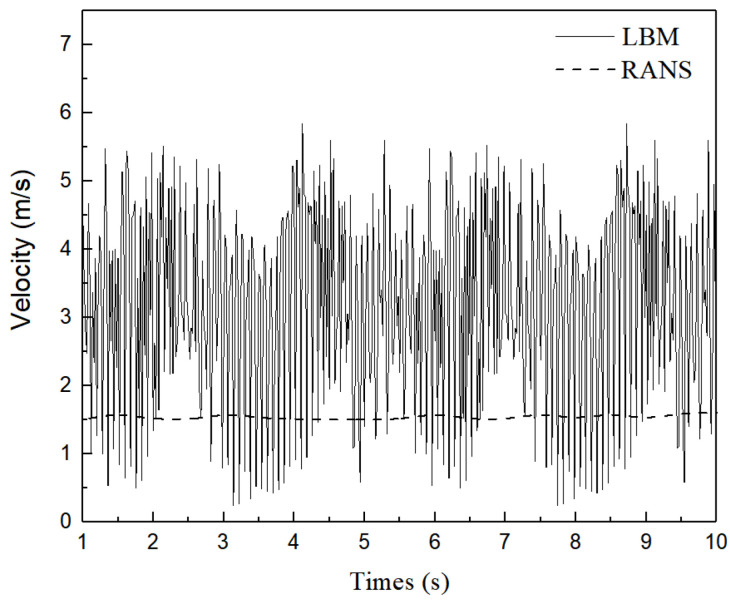
Comparison between velocity time series at Point 1 using the LBM and RANS models.

Transient fluid-particle flow within the SEN is reproduced, the oscillation of fluid-particle flow is quantitatively analysed in this section. The periodic behavior within the SEN will be characterised mathematically here. The velocity magnitude fluctuations at monitoring Points 1 and 2, located at the upper corner and the middle along the exit port of the SEN (Figure 1), respectively. Figure 12 shows the comparison between the velocity time series at Point 1 using different models. Numerical simulations using the RANS (*k*-*ε* turbulent) model are conducted to compare the transient flow within the SEN through LBM model. The solid bold lines correspond to simulations using the LBM model, and dotted lines represent the RANS model. The RANS results illustrate that the velocity fluctuations at the exit port are relatively stable, which are not as severe as those obtained from the LBM results. The behavior of mesoscopic fluid-particles inside the SEN exhibits periodic changes.

**Figure 13:**
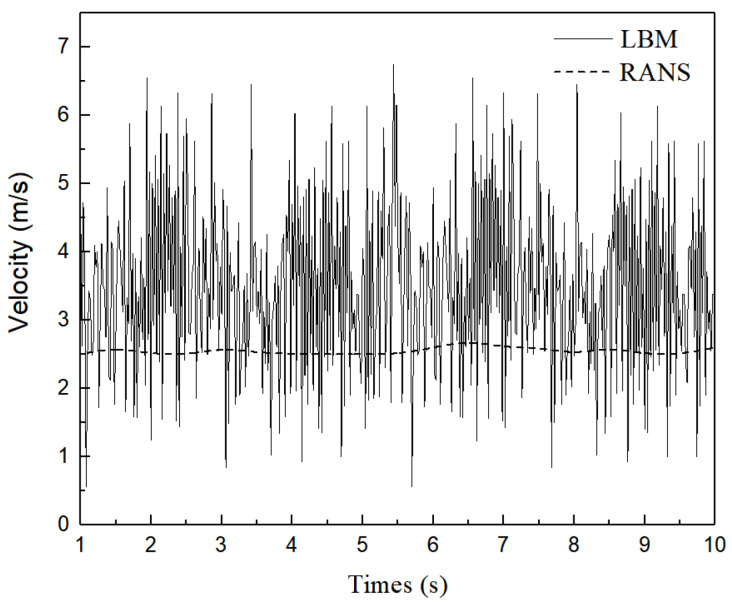
Comparison between velocity time series at Point 2 using LBM and RANS models.

All numerical simulations presented are performed under the same the conditions. Figure 13 demonstrates the velocity change for Point 2 through the LBM and RANS models. In the simulation of the RANS model, the dynamic change in the SEN is not evident; however, the LBM simulation also demonstrates that the flow behavior in periodic variation predicts a greater velocity oscillation close to the port (Point 2) than one at the corner (Point 1) within the SEN, which promotes the oscillations of the jet thereon. From the above comparison, the results show that the flow oscillations initially occur on the interior bifurcation tip due to flow separation, where the area suddenly changes in the shape of SEN geometry. The oscillations of the jet are intensified when the internal flow develop along the exit port of the SEN.

### 4.2. Flow Structures

**Figure 14:**
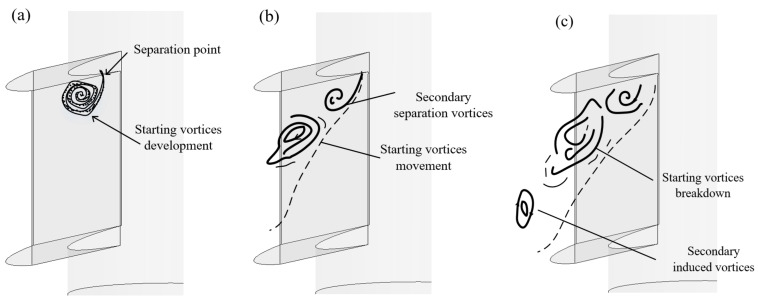
Formation, development, and dissipation of the vortices at the channel of the SEN at different stages: (**a**) formation of the vortices, (**b**) development of the vortices, and (**c**) dissipation of vortices.

The vortex structures play a significant role in the flow pattern on both sides of the SEN. To clarify this, the process of vortices development within the SEN is schematically divided into four stages, as shown in Figure 14a–c:

(I) Formation of the vortices: the vortices emerge at the upper corner of the SEN. The area of a flow suddenly increases in channels, and separate at the boundary layer;
(II) Development of the vortices: the size of the vortices increases gradually. The vortices develop along with the exit port and produce more vortices inside the SEN;
(III) Dissipation of vortices: the vortices interact with each other and disappear inside the SEN. The vortices persist for a relatively short period.

**Figure 15:**
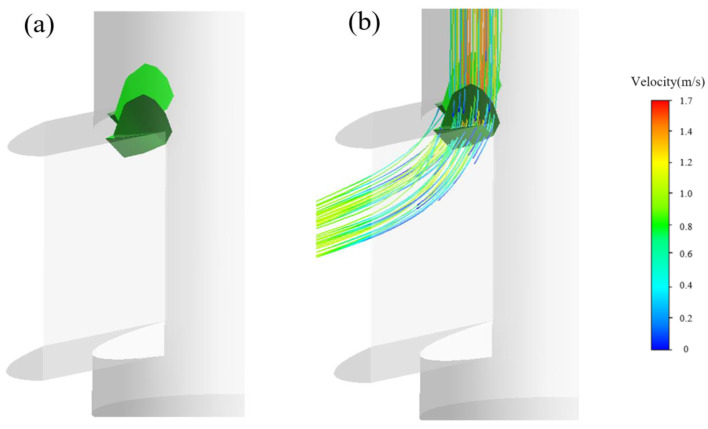
Formation of vortex structures (**a**) at the interior corner of the SEN port and (**b**) corresponding streamlines.

**Figure 16:**
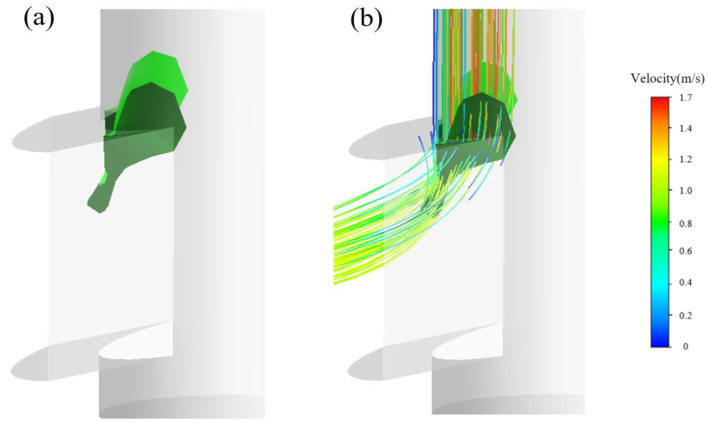
Development of vortex structures (**a**) at the upper edge of the SEN port (**b**) corresponding streamlines.

**Figure 17:**
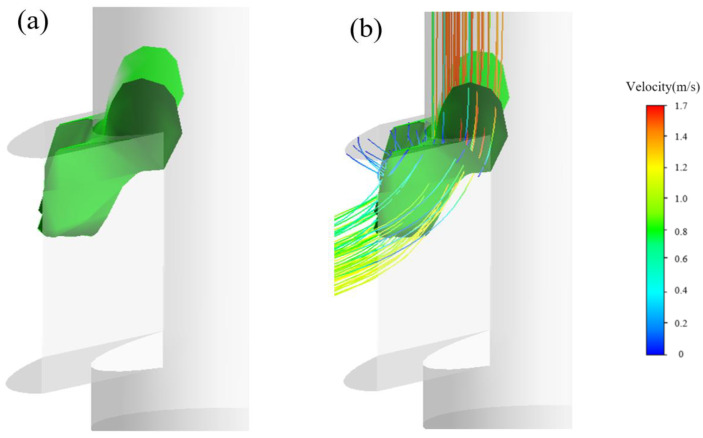
Diffusion of vortices (**a**) along with the channel of SEN port and (**b**) corresponding streamlines.

**Figure 18:**
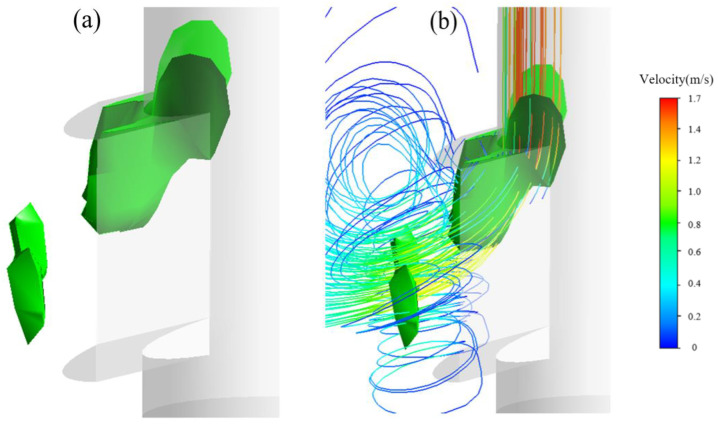
Separation of vortex structures (**a**) nearby the SEN port and (**b**) corresponding streamlines as well as accompanying swirling flow.

Differing from those studies, in this simulation, we propose that the evolution of the vortex structures is induced in terms of satisfaction of the *Q*-criterion. Figure 15, Figure 16, Figure 17 and Figure 18 show the vortices induced by the *Q*-criterion and corresponding streamlines. To illustrate the evolution of the vortices, Figure 15 illustrates that the vortices with the greatest strength are mainly concentrated at the upper corner of the SEN. The intensities (driving force) of the vortices are relatively large, which are of great importance to the evolution of vortex structures. Next, the corresponding vortices propagate from the upper corner to the exit port of the SEN, as shown in Figure 8. After that, the vortices then spread and deform along with the exit port, as shown in Figure 16 and Figure 17. The small vortices are finally separated from their larger counterparts close to the exit port, increasing the instability of the jet flow from the exit port. In addition, the separated vortices are also found (Figure 18), accompanied by small swirling flows, which are consistent with the phenomenon of the “stepped” jets that are observed in a water experiment.

**Figure 19:**
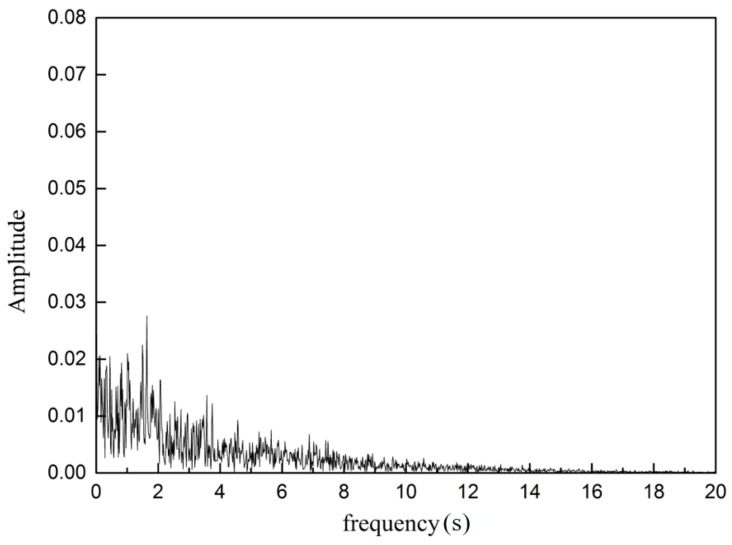
Power spectrum of velocity magnitude inside the exit point at Point 1.

**Figure 20:**
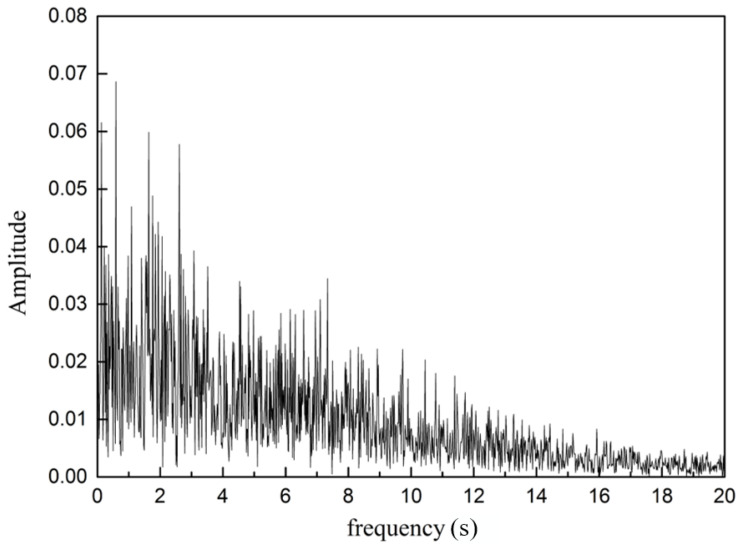
Power spectrum of velocity magnitude inside the exit point at Point 2.

To investigate the oscillating flow, the velocity time series obtained from the LBM model is processed into a power spectrum to estimate the oscillation period of the circulation. These samples are then processed into a power spectrum by using a fast Fourier transform (FFT) routine as defined by [25]:

(13) $$P\left(f_{k}\right)=\frac{1}{N^{2}}{\left|f_{k}\right|}^{2}\;k=1,\;2,\ldots ,\;N/2$$

(14) $$U_{k}=\sum_{}^{N}ue^{2\pi ijk/N}\;k=1,\;2,\ldots ,\;N$$

(15) $$f_{k}=\frac{k}{N\Delta t_{N}}\;k=1,\;2,\ldots ,\;N/2$$

where *u* is the velocity in the time series, *t_N_* denotes the times at the start and the end of the sampling period; *N* represents the number of sampling points. Thus, a frequency analysis of the velocity magnitude time series is conducted. In order to study the oscillating flow along the exit port of the SEN, frequencies of velocity fluctuation are analyzed at monitoring points P1, and P2 (Figure 1). Figure 19 and Figure 20 show the power spectrum of velocity magnitude with the SEN port at the corner (Point 1) and the exit port (Point 2), respectively. The dominant peak represents the oscillation frequency. There are many frequencies in the flow at the SEN exit port. The oscillations are not a simple combination of modes inside the SEN. The maximum amplitude is 0.03 at frequency 1 Hz at Point 1, however, this exceeds 0.07 at Point 2. It can be concluded that the flow oscillations have been intensified close to the exit port of the SEN.

## 5. Conclusions

The fluid-particle flow inside a bifurcated SEN is studied to evaluate flow oscillations through the LBM model. From the results, the following conclusions are drawn:

(1) The accuracy of the model has been verified by comparing vortex structures and simulated velocities with published experimental values inside and outside the SEN. Comparing simulations with RANS and LBM models, the results show that the mesoscopic LBM can predict the transient fluid-particle flow along the exit port of the SEN in detail, which can better reproduce the behavior of the oscillating jet within the SEN.
(2) The transient flow behavior results from the dynamic interaction of fluid-particle flow within the SEN. The velocities always exhibit strong high-frequency components. The periodic changes of mesoscopic fluid-particles flow within the SEN can be reproduced even under stable operating conditions. The visualization of internal flow within the SEN show that the LBM–LES–DPM coupled model is good at predicting the transient vortical flow within the SEN.
(3) Vortex structures are of great importance to the development of turbulent flow along the exit port of the SEN. The turbulent viscosity of flow near exit output of the SEN always fluctuates at both sides of the exit port. The asymmetric viscosities at both sides become similarly mirror-like at different times. The formation, development, and dissipation of detached vortices induced by the *Q*-criterion that identifies vortex structures can cause oscillation of jet within the SEN.
(4) There are many frequencies (between 1 Hz and 3 Hz) seen in flow oscillations along the channel of the SEN port. The oscillations are not a simple combination of modes and flow oscillations have been intensified simultaneously. The jet oscillations must originate from within the SEN interior and are intensified along the exit port.

This work represents our further efforts towards fluid-particles flow, vortices structures and their effects on turbulence inside bifurcated SEN flow. Further studies are needed to study the behaviour of introduced particles or bubbles under the framework of LBM model, the interaction between vortex structure and bubbles as well as their interactions (aggregation and breakdown).

## Figures and Tables

**Table 1:** Simulation conditions considered in the model.

Parameter	Value
Mold width (m)	1.2
Mold thickness (m)	0.23
Casting speed (m·min^−1^)	1.3
SEN submergence depth (mm)	200
SEN port angle (deg)	15°
SEN port shape	Rectangle
SEN port height (mm)	45
SEN port width (mm)	35
Molten steel density (kg·m^−3^)	7000
Molten steel viscosity (Pa·s)	0.006

## Data Availability

Not applicable.
